# *Ins1*^Cre^ knock-in mice for beta cell-specific gene recombination

**DOI:** 10.1007/s00125-014-3468-5

**Published:** 2014-12-11

**Authors:** Bernard Thorens, David Tarussio, Miguel Angel Maestro, Meritxell Rovira, Eija Heikkilä, Jorge Ferrer

**Affiliations:** 1 Center for Integrative Genomics, University of Lausanne, Genopode Building, CH-1015 Lausanne, Switzerland; 2 Institut d’Investigacions Biomèdiques August Pi i Sunyer (IDIBAPS), Barcelona, Spain; 3 Centro de Investigación Biomédica en Red de Diabetes y Enfermedades Metabólicas Asociadas (CIBERDEM), Spain, http://www.ciberdem.org/; 4 Department of Medicine, Imperial Centre for Translational and Experimental Medicine, Imperial College London, London, W12 0NN UK

**Keywords:** Beta cells, Cre recombinase, Glucose homeostasis, Hypothalamus, Insulin, Pancreatic islets, Transgenic mice

## Abstract

**Aims/hypothesis:**

Pancreatic beta cells play a central role in the control of glucose homeostasis by secreting insulin to stimulate glucose uptake by peripheral tissues. Understanding the molecular mechanisms that control beta cell function and plasticity has critical implications for the pathophysiology and therapy of major forms of diabetes. Selective gene inactivation in pancreatic beta cells, using the Cre-lox system, is a powerful approach to assess the role of particular genes in beta cells and their impact on whole body glucose homeostasis. Several Cre recombinase (Cre) deleter mice have been established to allow inactivation of genes in beta cells, but many show non-specific recombination in other cell types, often in the brain.

**Methods:**

We describe the generation of *Ins1* ^Cre^ and *Ins1* ^CreERT2^ mice in which the Cre or Cre-oestrogen receptor fusion protein (CreERT2) recombinases have been introduced at the initiation codon of the *Ins1* gene.

**Results:**

We show that *Ins1* ^Cre^ mice induce efficient and selective recombination of floxed genes in beta cells from the time of birth, with no recombination in the central nervous system. These mice have normal body weight and glucose homeostasis. Furthermore, we show that tamoxifen treatment of adult *Ins1* ^CreERT2^ mice crossed with *Rosa26-tdTomato* mice induces efficient recombination in beta cells.

**Conclusions/interpretation:**

These two strains of deleter mice are useful new resources to investigate the molecular physiology of pancreatic beta cells.

**Electronic supplementary material:**

The online version of this article (doi:10.1007/s00125-014-3468-5) contains peer-reviewed but unedited supplementary material, which is available to authorised users.

## Introduction

Pancreatic beta cells participate in glucose homeostasis by secreting insulin in response to rises in glycaemia to stimulate glucose uptake by peripheral tissues. To maintain this capacity over a lifetime, the insulin secretion capacity of individual beta cells as well as their number may be modulated to compensate for the development of insulin resistance in target tissues. Failure of this beta cell adaptive capacity is a major cause of the development of diabetic hyperglycaemia. On the other hand, the enhancement of natural mechanisms of beta cell adaptation might prove useful for regenerative therapies in autoimmune type 1 diabetes. Understanding the molecular basis of beta cell plasticity is therefore a central focus in diabetes research [1].

Genetically modified mice are being used extensively to study the role of specific genes in diabetes pathophysiology [2–5], and several genetic systems have been developed to overexpress or inactivate genes selectively in beta cells [6–12]. In particular, recent recombinant technologies have provided the possibility to selectively inactivate genes of interest in a tissue- and time-dependent manner, as critically reviewed recently [13]. A particularly useful approach consists in flanking the DNA sequence to be deleted by tandemly oriented 34 nucleotide-long *lox* sequences, which are recognised by Cre recombinase (Cre), a recombination enzyme that leads to the elimination of the sequence present between the two *lox* sites. Modification of the target DNA sequence with *lox* sites is performed by homologous recombination in embryonic stem cells, and the mice that are ultimately generated are crossed with mice that express Cre under a tissue-specific promoter to allow tissue-specific gene deletion. The use of Cre-oestrogen receptor fusion protein (CreERT2) [14], which is Cre fused to a modified form of the ligand-binding domain of the human oestrogen receptor that can be activated by tamoxifen, allows precise time-controlled genetic recombination.

One key aspect of the Cre-lox system is that transgenic expression of the *Cre* gene must be specific to the desired target tissue. Deletion in beta cells has been performed using mice expressing *Cre* under the control of various promoters such as that of the rat *Ins2* gene [12, 15], the mouse *Ins1* promoter [16] or the human insulin promoter [17]. In contrast to the mouse *Ins2* gene, which is also expressed in the embryonic and adult brain, the *Ins1* gene is selectively expressed by the beta cells [18, 19], thereby providing a potential specificity advantage to drive Cre reporter expression only in beta cells. Existing transgenics that use insulin gene regulatory regions typically allow for beta cell gene inactivation; however, they often also lead to expression in brain regions, mostly the hypothalamus for the *Ins2* promoter, leading to phenotypes that are complicated by the non-specific site of expression of the recombinase. Other approaches have used the promoters of transcription factor genes expressed at different stages of beta cell development, such as *Pdx-1*, *Neurog3*, *Nkx2.2*, *Pax4*, *Pax6* or *Isl-1* (for detailed review of these lines, see [13]). Although all of these lead to Cre expression in beta cells, they also lead to gene deletion either in islet non-beta cells or diverse non-pancreatic cellular lineages, including widely used *Pdx1-Cre* mouse lines that induce recombination in the exocrine pancreas. There is thus a need for more selective Cre lines for gene inactivation in beta cells.

The design of a mouse model that transcribes *Cre* exclusively in beta cells faces several obstacles. One is that gene regulatory sequences are not exclusively encoded in the 5′ regions of genes but are often determined or modulated by long-range *cis*-regulatory interactions, most of which have not been annotated. This obstacle is best addressed by inserting the recombinase in the endogenous genomic locus of interest, rather than using predefined promoter fragments. Another complexity is that most genes that are enriched in beta cells are also expressed in other cell types, some of which are central to glucose homeostasis. Notably, of the two non-allelic mouse insulin genes, *Ins2* is the most abundantly expressed in beta cells, yet it is also expressed in hypothalamus and brain [18]. Thus, even genetic models that succeed in recapitulating *Ins2* expression will unavoidably lead to expression outside the beta cells, while recapitulation of *Ins1* expression is expected to lead to selective beta cell expression

Here, we generated mice in which *Cre* was introduced at the initiator codon of the *Ins1* locus by homologous recombination technology so as to ensure faithful expression of the recombinase in beta cells. We show that this approach allowed for highly efficient expression of Cre or CreERT2 recombinase in beta cells and that the specificity of expression was guaranteed with no expression in hypothalamus or other brain areas and with recombination as early as the time of birth.

## Methods

**Fig. 1:**
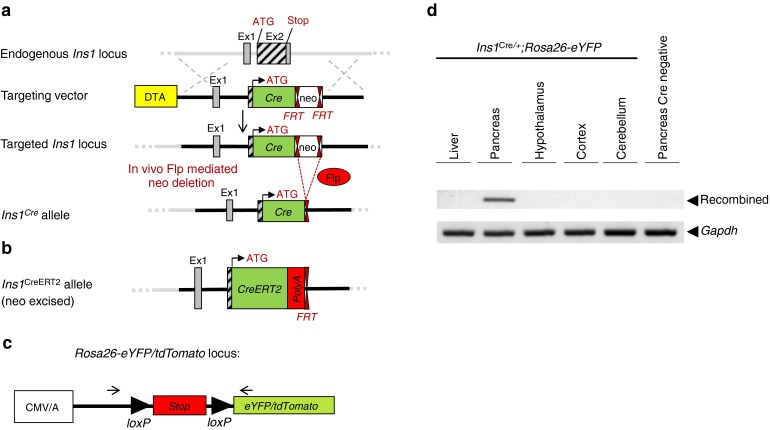
Generation of Ins1^*Cre*^ and *Ins1* ^CreERT2^ mice. (**a**) Structure of the *Ins1* locus, of the targeting vector, of the targeted allele, and of the *Ins1* ^Cre^ locus following flipase (Flp)-dependent removal of the flipase recognition site (Frt)-flanked neo cassette. DTA, diphtheria toxin receptor gene; Ex, exon; ATG, translation initiation codon. (**b**) The *Ins1* ^CreERT2^ allele was obtained using the targeting strategy depicted in (**a**). (**c**) Structure of the *Rosa26-eYFP/tdTomato* transgene and localisation of the primers (arrows) used for PCR analysis of the recombined locus. CMV/A, cytomegalovirus/actin promoter; (**d**) PCR analysis shows recombination in the pancreas but not in the liver, cortex, hypothalamus or cerebellum of *Ins1* ^Cre/+^ *;Rosa26-eYFP*. No recombination was observed in the pancreas of *Rosa26-eYFP* littermates; amplification of the *Gapdh* gene is used as a control

*Mice Ins1* ^Cre^ (*Ins1* ^tm1(cre)Thor^) and *Ins1* ^CreERT2^ (*Ins1* ^tm1(CreERT2)Thor^) knock-in mice were generated by Genoway (Lyon, France) and kept on a C57Bl/6J genetic background. Briefly, a targeting vector was created by inserting the *Cre* or *CreERT2* recombinase genes by homologous recombination in the second exon of the *Ins1* gene so that the coding region of the recombinase starts at the initiation codon and replaces the *Ins1* coding sequence (Fig. 1). Following transfection of the targeting vector into embryonic stem cells, negative and positive selections were performed by diphtheria toxin and neomycin (neo) selection, respectively. Cells with confirmed homologous recombination were used to generate chimeric mice. These were crossed with C57Bl/6J mice, and mice with germline transmission were crossed with C57Bl/6J flippase (Flp) deleter mice to eliminate the neo cassette. Mice with germline transmission of the recombined allele and deletion of the neo cassette were then crossed with *Rosa26-eYFP* mice [20] or *Rosa26-tdTomato* mice [21]. Animals were housed four to five per cage at 23°C on a 12 h light/dark cycle and were fed a standard rodent chow (Diet 3436*;* Provimi Kliba, Penthallaz, Switzerland). To induce recombination in *Ins1* ^CreERT2^ crossed with *Rosa26* reporter mice, tamoxifen (T5648; Sigma-Aldrich, St Louis, MO, USA) was dissolved in corn oil at 20 mg/ml, and 2 mg was injected subcutaneously four times over a 2 week period.

### Genotyping

Mouse genotyping was performed by PCR analysis using primers listed in electronic supplementary material (ESM) Table 1.

### Histological and immunodetection procedures

For immunodetection of enhanced yellow fluorescent protein (eYFP), tdTomato, insulin, glucagon, pancreatic polypeptide and somatostatin in the pancreas or in the brain, mice were fixed by perfusion with cold 4% paraformaldehyde in sodium phosphate buffer (0.1 mol/l, pH 7.4). The organs were dissected and kept for 4 h in the same paraformaldehyde solution before overnight incubation in 30% sucrose, then frozen in isopentane and stored at −80°C until used.

For immunofluorescence detection studies in eYFP reporter mice 20 μm cryosections were preincubated for 30 min in a permeabilisation blocking buffer (0.1 mol/l PBS, pH 7.4, 3% BSA A8022, 0.3% Triton X-100, 9002-93-1; Sigma-Aldrich), then incubated overnight at 4°C with a rabbit polyclonal antibody to green fluorescent protein (GFP) (ab290, diluted 1:2,000; Abcam, Cambridge, UK), a polyclonal guinea pig anti-insulin (AO564, diluted 1:400; Dako, Baar, Switzerland) or anti-glucagon (4031-01F, diluted 1:500; Linco Research, St Charles, MO, USA), a polyclonal anti-pancreatic polypeptide (diluted 1:1,000; Linco Research) or a polyclonal rabbit anti-somatostatin (A0566, diluted 1:200; Dako). After washing, the sections were then incubated for 90 min with Alexa Fluor 488 goat anti-guinea pig IgG (H+L) (A11073, diluted 1:200; Invitrogen, LuBioScience, Lucerne, Switzerland) or with a goat anti-rabbit immunoglobulin-Cy3 conjugate (111-165-144, diluted 1/100; Jackson ImmunoResearch, West Grove, PA, USA), or for the *Rosa26-tdTomato* mice with Alexa Fluor 488 goat anti-rabbit IgG (H+L) (A-11008, diluted 1:200; Invitrogen). Immunofluorescence analysis of tdTomato was carried out in 3 μm paraffin sections, essentially as described [22, 23], using a rabbit polyclonal anti-red fluorescent protein (600-401-379; Rockland Immunochemical, Gilbertsville, PA, USA).

For enzymatic detection of eYFP, after overnight incubation with primary antibody, sections were washed and incubated for 90 min with a biotinylated goat anti-rabbit antibody (BA-1000, diluted 1:750; Vector Laboratories, Burlingame, CA, USA), incubated for 30 min with a biotin–avidin complex (Vector) and stained using 3,3′-diaminobenzidine tetrahydrochloride (Sigma-Aldrich).

Sections were observed using an Axio Imager D1 microscope (Carl Zeiss, Oberkochen, Germany) interfaced with Axiovision software (Carl Zeiss) or a 510 Meta inverted confocal laser scanning microscope (LSM) with LSM software (release 3.5; Zeiss).

### Glucose tolerance tests

For glucose tolerance tests, 16 h fasted male and female mice of indicated ages were injected i.p. with glucose (2 g/kg), and blood glucose levels were determined from tail–tip bleedings at the indicated times relative to glucose injections. Blood glucose levels were measured using a glucometer (Ascensia Breeze 2; Bayer HealthCare, Switzerland).

### Statistical analysis

Data are expressed as means±SD or SEM. Statistical analysis was performed using an unpaired two-tailed Student’s *t* test. Values of *p* < 0.05 were considered significant.

### Study approval

All breeding and cohort maintenance performed in our animal facility and all experiments were approved by the Service Vétérinaire du Canton de Vaud and the Comitè Ètic d’Experimentació Animal of the University of Barcelona.

## Results

### Mouse generation

Generation of the *Ins1* ^Cre^ and *Ins1* ^CreERT2^ mice followed the strategy described in Fig. 1a–c and the Methods section, allowing expression of Cre or CreERT2 from the translation initiation codon of the *Ins1* gene. Embryonic stem cells with homologous recombination were used to generate chimeric mice. These were crossed with Flp deleter mice to remove the neo cassette. The resulting *Ins1* ^Cre^ mice were crossed with *Rosa26-eYFP* mice to determine the anatomical sites of Cre-dependent *lox* site recombination. PCR analysis of the recombined *Rosa26-eYFP* locus (Fig. 1c) indicated recombination in whole pancreas DNA preparations but not in DNA extracted from liver, hypothalamus, cortex or cerebellum (Fig. 1d).

**Fig. 2:**
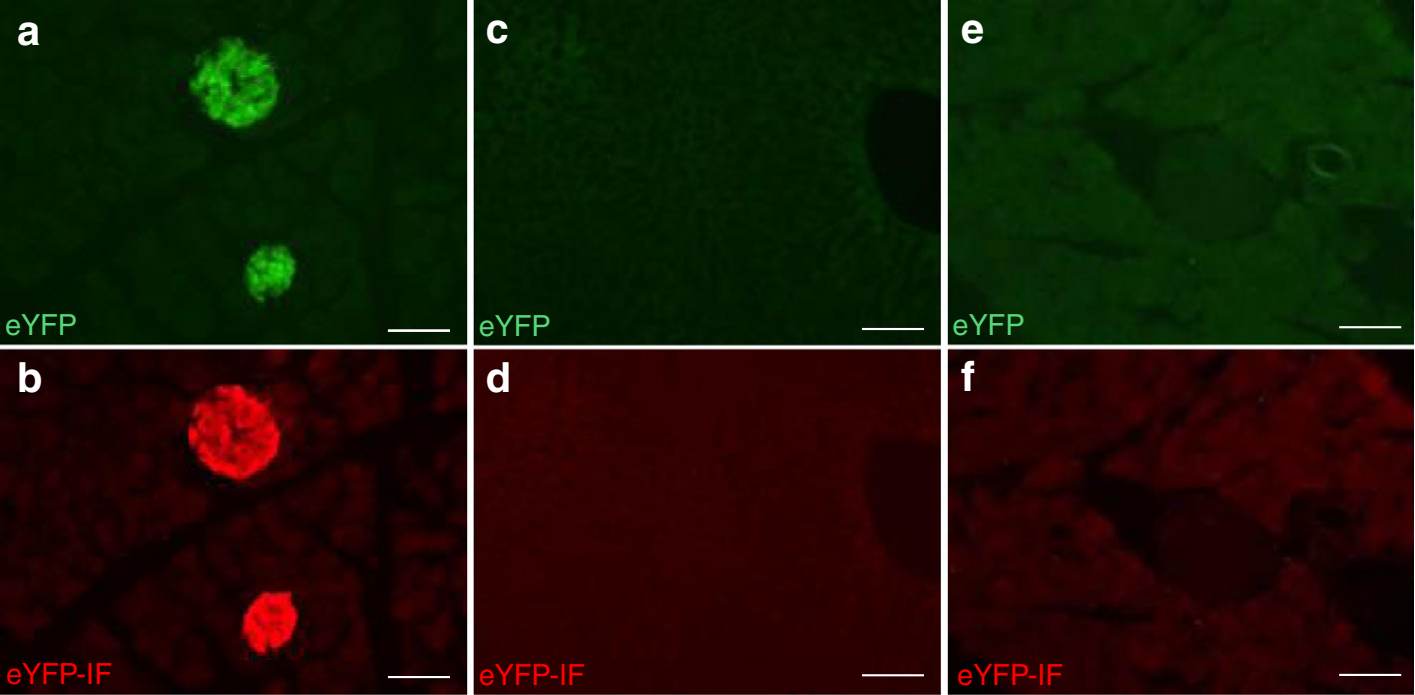
Expression of eYFP in pancreatic islets of *Ins1* ^Cre/+^ *;Rosa26-eYFP* mice. (**a**, **c**, **e**) eYFP fluorescence is detected in the pancreatic islets (**a**) but not in the liver (**c**) of 12-week-old *Ins1* ^Cre/+^ *;Rosa26-eYFP* mice; it is not detected in the pancreas of *Rosa26-eYFP* mice (**e**). (**b**, **d**, **f**) Indirect immunofluorescence detection of eYFP (eYFP-IF, red) on tissue sections corresponding to **a**, **c** and **e**, respectively. Scale bars, 50 μm (**a**–**f**)

**Fig. 3:**
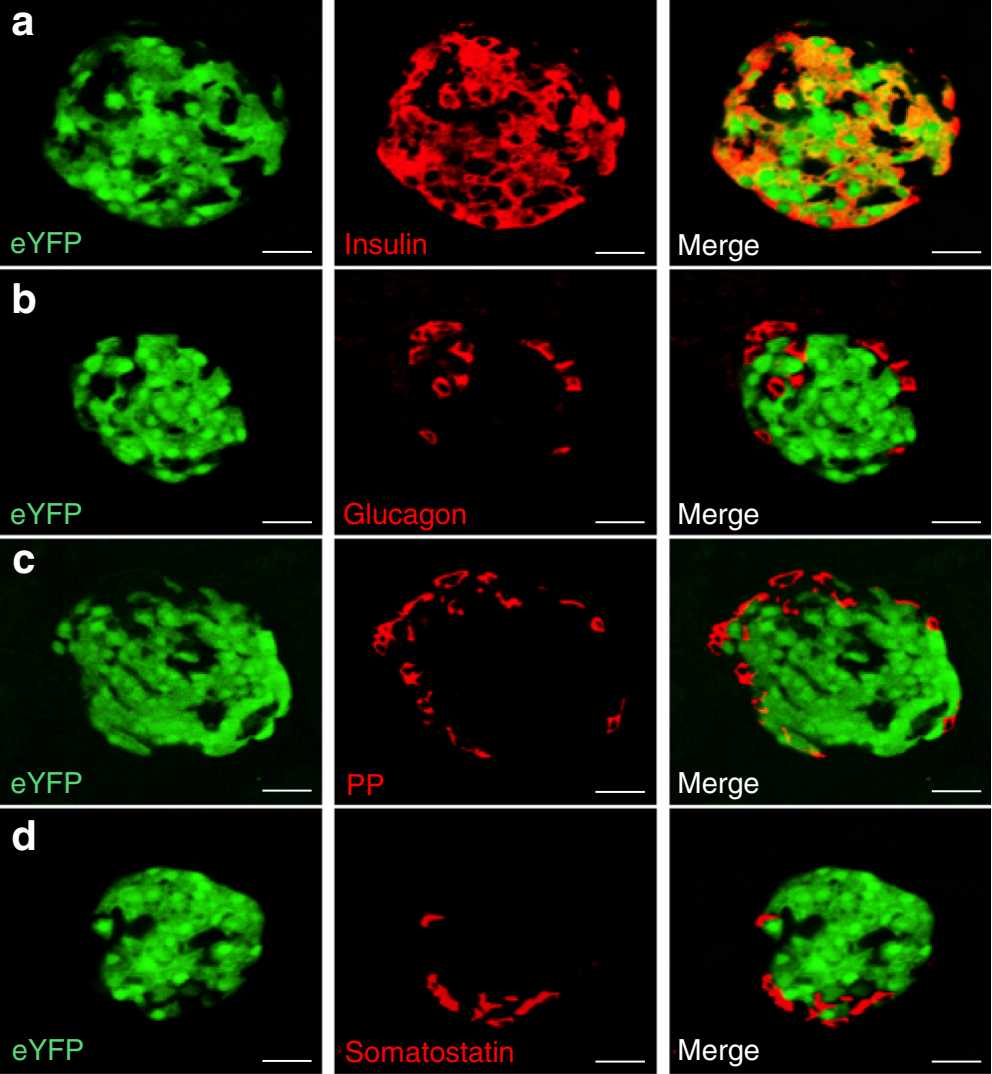
Expression of eYFP is restricted to beta cells of *Ins1* ^Cre/+^ *;Rosa26-eYFP* mice. eYFP expression in *Ins1* ^Cre/+^ *;Rosa26-eYFP* mouse islets co-stained for insulin (**a**), glucagon (**b**), pancreatic polypeptide (PP) (**c**) and somatostatin (**d**). Scale bars, 20 μm (**a**–**d**)

Assessment of the cellular sites of Cre-induced eYFP expression was then performed by histological analysis. In the pancreas, eYFP expression was only observed in islets as revealed by the endogenous fluorescence of eYFP or by immunofluorescence detection using anti-GFP antibodies (Fig. 2a, b). No eYFP expression was observed in the liver of the same mice (Fig. 2c, d), nor in the pancreas of control *Rosa26-eYFP* mice (Fig. 2e, f). Co-staining of the pancreas of *Ins1* ^Cre/+^ *;Rosa26-eYFP* mice with islet hormones (insulin, glucagon, somatostatin or pancreatic polypeptide) showed that eYFP expression was specific to beta cells within pancreatic islets (Fig. 3a–d). We quantified the recombination efficiency in pancreatic cell subtypes using *Ins1* ^Cre/+^ *;Rosa26-tdTomato* mice and found that 97.8 ± 1.2% of insulin-expressing cells showed expression of tdTomato, in contrast with 3.0 ± 0.3% of glucagon-expressing cells, 0.0% of somatostatin-expressing cells and <0.01% of CPA-1-expressing pancreatic acinar cells.

**Fig. 4:**
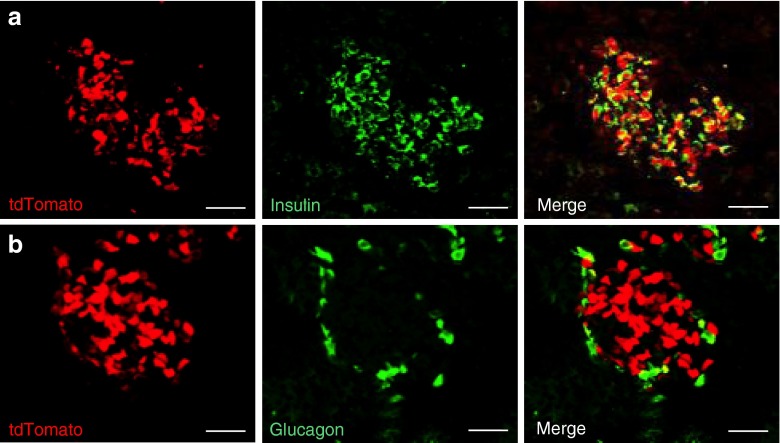
Expression of eYFP in beta cells of neonatal *Ins1* ^Cre/+^ *;Rosa26-tdTomato* mice. (**a**) Expression of tdTomato (red) and co-staining for insulin (green) in the islet of 1-day-old *Ins1* ^Cre/+^ *;Rosa26-eYFP* mice. (**b**) Expression of tdTomato (red) and co-staining for glucagon (green). Scale bars, 20 μm (**a**, **b**)

To determine whether recombination could already be seen at the time of birth, pancreas sections were prepared from newborn *Ins1* ^Cre/+^ *;Rosa26-tdTomato* mice and co-stained for insulin and glucagon. Figure 4a, b shows that expression of tdTomato was already present in beta cells at birth and no expression was detected in glucagon-producing alpha cells.

**Fig. 5:**
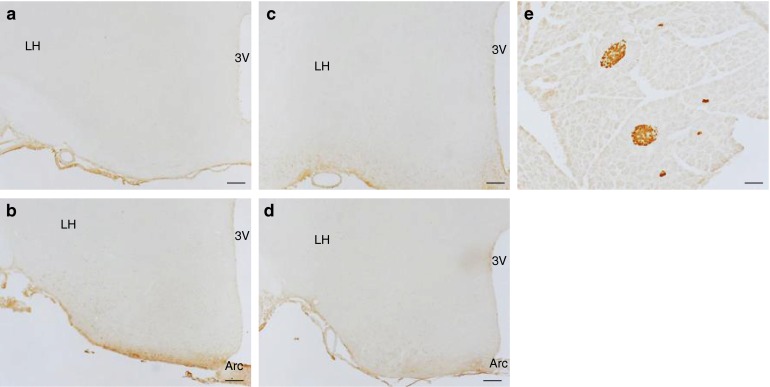
No expression of eYFP in the hypothalamus of *Ins1* ^Cre/+^ *;Rosa26-eYFP* mice as assessed by immunohistochemistry. Immunohistochemical detection of eYFP in the hypothalamus of *Ins1* ^Cre/+^ *;Rosa26-eYFP* and control *Rosa26-eYFP* mice at bregma −0.82 (**a**, **c**) and −1.70 (**b**, **d**). (**e**) eYFP immunohistochemical detection in the pancreas from *Ins1* ^Cre/+^ *;Rosa26-eYFP* mice*.* Scale bars, 100 μm. Arc, arcuate hypothalamic nucleus; LH, lateral hypothalamic area; 3 V, third ventricle

Because Cre mice previously generated to target the pancreatic beta cells often showed expression of the transgene in the brain, in particular in the hypothalamus, we verified expression of eYFP in different parts of the brain. PCR analysis (Fig. 1d) showed no detectable recombination of the *Rosa26* locus in *Ins1* ^Cre/+^ *;Rosa26-eYFP* mice in the hypothalamus, cortex or cerebellum. No eYFP fluorescence could be detected in the hypothalamus of *Ins1* ^Cre/+^ *;Rosa26-eYFP* mice, as shown at two different bregma levels (ESM Fig. 1a, b); sections of the hypothalamus of control *Rosa26-eYFP* mice are shown in ESM Fig. 1c, d. As an alternative means of detecting recombination in the brain, we evaluated expression of eYFP by immunohistochemistry. No signal could be detected in any hypothalamic areas of *Ins1* ^Cre/+^ *;Rosa26-eYFP* mice, as shown in Fig. 5a–d. Immunohistochemical detection of eYFP in the beta cells from *Ins1* ^Cre/+^ *;Rosa26-eYFP* mice is shown as a positive control (Fig. 5e). Similarly, no positive signals could be detected in the cortex or thalamus of *Ins1* ^Cre/+^ *;Rosa26-eYFP* mice (ESM Fig. 2a–d). Thus, these data are in agreement with the PCR data of Fig. 1c that show absence of recombination event in the brain of adult *Ins1* ^Cre/+^ *;Rosa26-eYFP* mice.

**Fig. 6:**
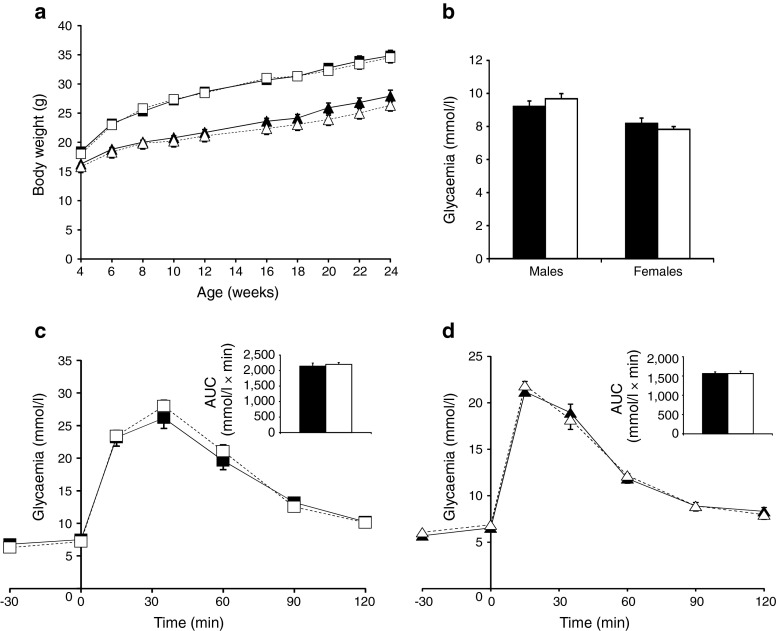
Normal body weight gain, glycaemic levels and glucose tolerance in heterozygous *Ins1* ^Cre^ mice. (**a**) Body weight of male (white squares) and female (white triangles) *Ins1* ^Cre^ mice and their control littermates (males, black squares; females, black triangles). (**b**) Fed glycaemic levels in 12-week-old male and female *Ins1* ^Cre^ mice and their control littermates. (**c**, **d**) Glucose tolerance tests in 12-week-old male (**c**) and female (**d**) *Ins1* ^Cre^ mice and their control littermates (white symbols, *Ins1* ^Cre^ mice; black symbols, control littermates). White bars, *Ins1* ^Cre^ mice; black bars, control littermates. Data are mean±SEM; *n* = 9–13

To determine whether introduction of the *Cre* gene in the *Ins1* locus would cause any deregulation of glucose homeostasis or impact mouse growth, we generated cohorts of male and female C57Bl/6J heterozygous *Ins1* ^Cre^ and littermate controls and followed them for 24 weeks. No difference in body weight gain could be observed between the control and *Ins1* ^Cre^ mice of either sex (Fig. 6a). Random fed glycaemia levels were also identical (Fig. 6b), as was glucose tolerance assessed in heterozygous 12 week old male and female mice (Fig. 6c, d).

**Fig. 7:**
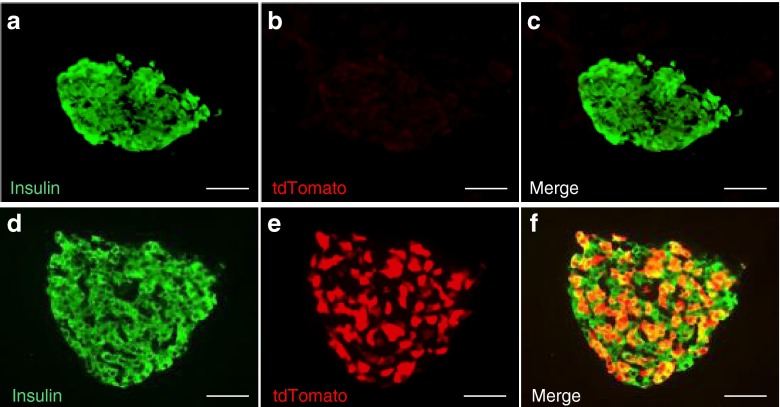
Tamoxifen-induced expression of tdTomato in beta cells of *Ins1* ^CreERT2/+^ *;Rosa26-tdTomato* mice. (**a–c**) Ten-week-old *Ins1* ^CreERT2/+^ *;Rosa26-tdTomato* mice were injected with 2 mg tamoxifen four times over a 2 week period. Expression of insulin (green) (**a**) and tdTomato (red) (**b**) was then assessed; (**c**) merged images. (**d–f**) Same as (**a–c**) except that mice were injected with vehicle (corn oil). Scale bars, 50 μm

Finally, to determine whether recombination could be induced in the beta cells of adult *Ins1* ^CreERT2/+^ *;Rosa26-tdTomato* mice, we tested several tamoxifen injection protocols. The data of Fig. 7 show that four injections of tamoxifen (2 mg in corn oil) over a 2 week period in 10 week old mice induced recombination in approximately 60–70% of beta cells. No expression of tdTomato could be observed in islets from corn oil-treated *Ins1* ^CreERT2/+^ *;Rosa26-tdTomato* mice.

## Discussion

Here we describe the generation of two new mouse models for efficient and selective induction of lox-dependent recombination in pancreatic beta cells. The *Ins1* ^Cre^ mice induce complete recombination by the time of birth in all beta cells, whereas the *Ins1* ^CreERT2^ mice can be used for tamoxifen-dependent gene recombination in adult mice.

Several *Cre* transgenic mice have already been established for beta cell-specific gene recombination. The present models use a knock-in strategy to express Cre from the endogenous *Ins1* locus. This locus is known to have a more restricted beta cell expression than the *Ins2* locus, which displays transient expression in the hypothalamus and other brain structures [24]. The use of the *Ins2* promoter to drive Cre expression led to mouse models with compound genetic recombination in the islets and brain, leading to complicated interpretation of the phenotype of mice used [16]. *Ins2* knock-in mouse lines have also been generated [25], but are similarly expected to recapitulate endogenous expression and result in ectopic expression in brain. On the other hand, two recent studies have reported transgenic models expressing Cre or CreER driven by *Ins1* promoter sequences or by an *Ins1* locus bacterial artificial chromosome [16, 26]. In keeping with our model, these mice lack Cre expression in the brain [16, 26]. Our model provides further advantages in that *Cre* is inserted in the native *Ins1* locus and is thus theoretically controlled by all known and unknown native *cis*-regulatory sequences, providing greater ability to recapitulate the endogenous *Ins1* expression pattern. We have indeed verified that recombination is efficiently induced in pancreatic islet beta cells at birth, whereas no recombination could be observed in the brain. Furthermore, we report an inducible *Ins1* ^CreERT2^ mouse line that is capable of recombination in 60–70% of the beta cells. Importantly, there was no leakiness of CreERT2 expression as judged by the absence of tdTomato expression in non-treated or vehicle-treated mice.

An important aspect of the *Ins1* ^Cre^ mouse model is that its glucose homeostasis and body weight control are indistinguishable from those of control littermates. It has been reported that one of the rat insulin promoter (RIP)-driven Cre mouse lines [11] has a defect in glucose homeostasis [27], casting some doubt on the results of reports showing an impact of beta cell gene deletion using this recombinase line. We have not tested body weight and glucose homeostasis in cohorts of *Ins1* ^CreERT2^ mice. However, because both the *Ins1* ^Cre^ and *Ins1* ^CreERT2^ mice were generated by the same knock-in strategy we anticipate similar effects. In the different transgenic mouse lines, such as RIP-Cre, functional differences may result from insertional mutagenesis caused by the random insertion of the transgene, usually as large concatemers. This can potentially lead to unwanted effects such as the formation of loss-of-function alleles, abnormalities in the activity of nearby genes, and unexpected expression pattern and strength of the transgene. The knock-in approach used here results in a well-defined targeted modification that is therefore more precise and less prone to induction of artefacts. Another theoretical consideration is that the *Ins2* promoter is stronger than *Ins1* [28] and could thus result in higher concentrations of Cre that are potentially deleterious. Importantly, our knock-in approach does not affect glucose homeostasis despite suppressing one *Ins1* allele, which is also in agreement with previous studies reporting perfect glycaemic control in mice with deletion of single alleles of the *Ins1* or *Ins2* locus [29].

In summary, we have generated two new Cre deleter mice that can be used for beta cell-specific constitutive (*Ins1* ^Cre^) or inducible (*Ins1* ^CreERT2^) deletion of genes. The constitutive deleter mice induce deletion in nearly all beta cells from the immediate postnatal period to adulthood and they display normal control of body weight and glycaemia. The inducible deleter mice will be useful to test the impact of gene deletion in adult mice or at a defined time during development, although deletion does not occur in all beta cells. These two mouse strains are therefore very valuable new tools for beta cell research.

## Electronic supplementary material

Below is the link to the electronic supplementary material.

ESM Fig. 1 (PDF 329 kb)

ESM Fig 2 (PDF 430 kb)

ESM Table 1 (PDF 66 kb)

## Abbreviations

Cre: Cre recombinase
CreERT2: Cre-oestrogen receptor fusion protein
eYFP: Enhanced yellow fluorescent protein
Flp: Flippase
GFP: Green fluorescent protein
Neo: Neomycin
RIP: Rat insulin promoter
