# Supplementary material for: Ins1Cre knock-in mice for beta cell-specific gene recombination
Source: Diabetologia. 2014 Dec 11;58(3):558–65. doi: 10.1007/s00125-014-3468-5 (PMC4320308; doi:10.1007/s00125-014-3468-5)
Supplement: Supplementary file 1 — (PDF 329 kb) [file 125_2014_3468_MOESM1_ESM.pdf]

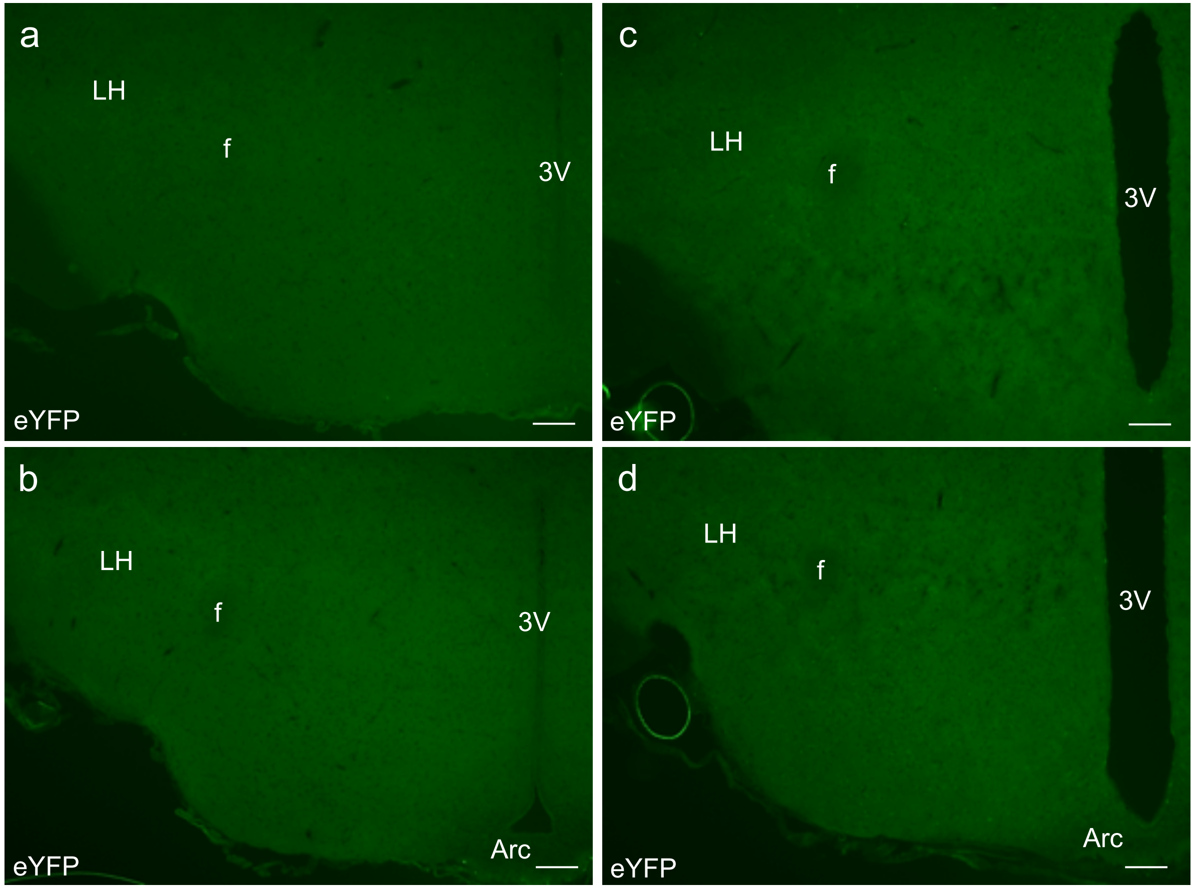

ESM Figure 1: No expression of eYFP in the brain of *Ins1*$^{Cre/+}$*;Rosa26-eYFP* mice. Hypothalamic section of *Ins1*$^{Cre/+}$*;Rosa26-eYFP* and control *Rosa26-eYFP* mice at Bregma -1.06 (a, c) and -1.58 (b, d). Scale bars: 100 µm (a-d). LH: Lateral Hypothalamic area, 3V: 3rd ventricle, Arc: Arcuate hypothalamic nucleus, f: fornix
