# Supplementary material for: Ins1Cre knock-in mice for beta cell-specific gene recombination
Source: Diabetologia. 2014 Dec 11;58(3):558–65. doi: 10.1007/s00125-014-3468-5 (PMC4320308; doi:10.1007/s00125-014-3468-5)
Supplement: Supplementary file 2 — (PDF 430 kb) [file 125_2014_3468_MOESM2_ESM.pdf]

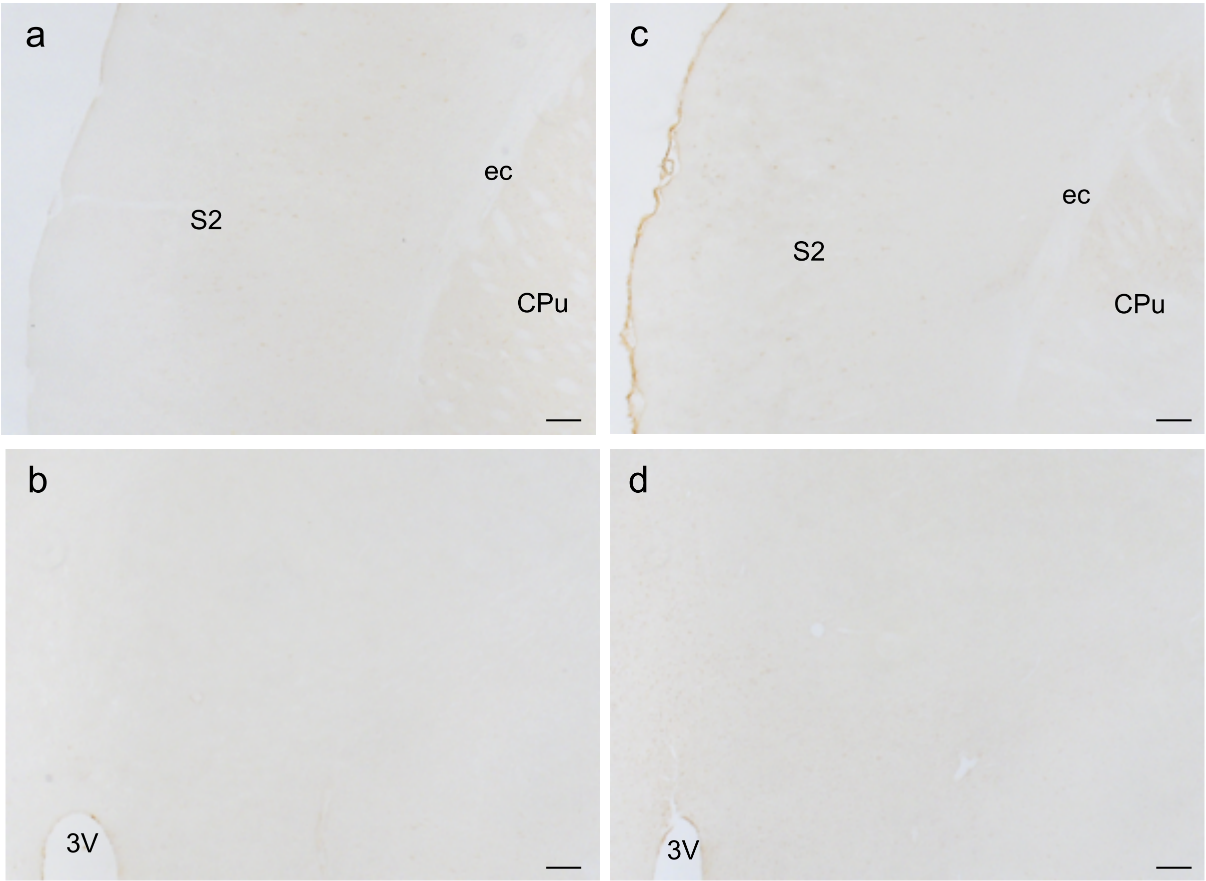

ESM Figure 2: No immunohistochemical detection of eYFP in the cortex and thalamus of *Ins1*$^{Cre/+}$*;Rosa26-eYFP* (a,b) or control *Rosa26-eYFP* mice (c,d). (a, c) cortex, Bregma -0.82. (b, d) Thalamus, Bregma -1.70. Scale bars: 100 µm (a-d). S2: 2nd somatic sensory cortex, ec: external capsule, CPu: caudate putamen, 3V: 3rd ventricle.
