# Supplementary material for: Ins1Cre knock-in mice for beta cell-specific gene recombination
Source: Diabetologia. 2014 Dec 11;58(3):558–65. doi: 10.1007/s00125-014-3468-5 (PMC4320308; doi:10.1007/s00125-014-3468-5)
Supplement: Supplementary file 3 — (PDF 66 kb) [file 125_2014_3468_MOESM3_ESM.pdf]

ESM Table 1: Oligonucleotide sequences used for allele genotyping

Oligonucleotide sequence	Purpose
5-AAACGTTGATGCCGGTGAACG-3	Genotyping *Cre*
5-CAGCCACCAGCTTGCATGAT-3	Genotyping *Cre*
5-AAAGTCGCTCTGAGTTGTTAT-3	Genotyping *eYFP*
5-GCGAAGAGTTTGTTCCTCAACC-3	Genotyping *eYFP*
5-GGAGCGGGAGGAATGGATATG-3	Genotyping *eYFP*
5’-AAGGGAGCTGCAGTGGAGTA-3’	Genotyping *tdTomato*
5’-CCGAAAATCTGTGGGAAGTC-3’	Genotyping *tdTomato*
5’-GGCATTAAAGCAGCGTATCC-3’	Genotyping *tdTomato*
5’-CTGTTCCTGTACGGCATGG-3’	Genotyping *tdTomato*
5-CAGTAGTCCAGGGTTTCCTTGATG-3	Genotyping recombined *Rosa26eYFP* alleles
5-ATAGGCACCACGCCGGTGAA-3	Genotyping recombined *Rosa26eYFP* alleles
5-GCCATCAACGACCCCTTCAT-3	*Gapdh*
5-TTCACACCCATCACAAACAT-3	*Gapdh*
